# Patient care in rapid-expansion intensive care units during the COVID-19 pandemic crisis

**DOI:** 10.1186/s12871-022-01752-z

**Published:** 2022-07-07

**Authors:** Jade I. Basem, Anna F. Roth, Robert S. White, Virginia E. Tangel, Silis Y. Jiang, Jacky M. Choi, Katherine L. Hoffman, Edward J. Schenck, Zachary A. Turnbull, Kane O. Pryor, Natalia S. Ivascu, Stavros G. Memtsoudis, Peter A. Goldstein

**Affiliations:** 1 Department of Anesthesiology, Weill Cornell Medicine, 1300 York Avenue, Room A-1050, NY 10065 New York, USA; 2 Department of Population Health Sciences, Division of Biostatistics and Epidemiology, Weill Cornell Medicine, New York, NY USA; 3 Department of Anesthesiology, Critical Care & Pain Management, Hospital for Special Surgery, New York, NY USA; 4 Department of Medicine, Weill Cornell Medicine, New York, NY USA; 5 Feil Family Brain & Mind Research Institute, Weill Cornell Medicine, New York, NY USA

**Keywords:** COVID-19, Critical care, Intensive care, ICU outcomes, Epidemiology

## Abstract

**Background:**

The coronavirus-2019 (COVID-19) pandemic highlighted the unfortunate reality that many hospitals have insufficient intensive care unit (ICU) capacity to meet massive, unanticipated increases in demand. To drastically increase ICU capacity, NewYork-Presbyterian/Weill Cornell Medical Center modified its existing operating rooms and post-anaesthesia care units during the initial expansion phase to accommodate the surge of critically ill patients.

**Methods:**

This retrospective chart review examined patient care in non-standard Expansion ICUs as compared to standard ICUs. We compared clinical data between the two settings to determine whether the expeditious development and deployment of critical care resources during an evolving medical crisis could provide appropriate care.

**Results:**

Sixty-six patients were admitted to Expansion ICUs from March 1^st^ to April 30^th^, 2020 and 343 were admitted to standard ICUs. Most patients were male (70%), White (30%), 45–64 years old (35%), non-smokers (73%), had hypertension (58%), and were hospitalized for a median of 40 days. For patients that died, there was no difference in treatment management, but the Expansion cohort had a higher median ICU length of stay (q = 0.037) and ventilatory length (q = 0.015). The cohorts had similar rates of discharge to home, but the Expansion ICU cohort had higher rates of discharge to a rehabilitation facility and overall lower mortality.

**Conclusions:**

We found no significantly worse outcomes for the Expansion ICU cohort compared to the standard ICU cohort at our institution during the COVID-19 pandemic, which demonstrates the feasibility of providing safe and effective care for patients in an Expansion ICU.

## Background

The Coronavirus-19 (COVID-19) pandemic, resulting from the coronavirus SARS-CoV-2, was first reported to have arrived in New York City (NYC) on March 1, 2020 [1]; the first reported NYC death occurred on March 14 [2]. As of 31 August 2021, the number of confirmed cases in NYC was 847,342, with 119,450 hospitalizations, and 28,664 confirmed deaths (https://www1.nyc.gov/site/doh/covid/covid-19-data.page).

Reports from China [3] and Europe [4] indicated wide clinical symptomatology—individuals could be asymptomatic carriers, mildly ill, to presenting with acute respiratory distress syndrome (ARDS). The high-degree of infectivity associated with COVID-19 (and the widespread presence of asymptomatic carriers) [5] likely contributed to the exponential growth seen during the pandemic’s early stages [6, 7]. The rapid increase in critically ill patients overwhelmed many hospitals and the United States, unfortunately, did not have critical care resources available to manage a crisis of this magnitude [8, 9]. With emergence of the highly transmissible Delta (B.1.617.2) variant of SARS-CoV-2 [10], global regions relatively spared from the first wave of infection in 2020 (*e.g*., Australasia) now face increasing stress on critical care resources [11, 12] due to the high rate of hospitalization associated with this variant [13, 14].

Anticipating a surge of critically ill COVID-19 patients, our hospital convened a multidisciplinary working group with representation from senior hospital administration, chiefs of service, nursing administration, and facilities management and engineering to consider how to efficiently increase our intensive care unit (ICU) capacity [15, 16]. Prior to the onset of the pandemic, there were a total of 109 adult intensive care unit (ICU) beds at NewYork-Presbyterian/Weill Cornell Medical Center (NYP-WCMC) distributed across a number of different care units: Burn (*n* = 15), Cardiac (*n* = 20), Cardiothoracic (*n* = 20), Medical (*n* = 20), Neurosurgical (*n* = 14), and Surgical/Post-Anaesthesia (*n* = 20).

It was determined that operating rooms (ORs) and post-anaesthesia care units (PACUs) were the most feasible locations for initial expansion (herein referred to as ‘Expansion-ICUs’) because of the available pre-existing infrastructure and personnel familiar with the majority of procedures commonly performed in critical care settings. At the peak of the pandemic, 60 Expansion-ICU beds were operational. During this time, all patients admitted to the traditional ICU and Expansion-ICU were intubated requiring mechanical ventilation.

During NYC’s COVID-19 spring 2020 surge, essentially all patients who were eligible for ICU care were intubated, thus, intubation was the prerequisite for our ICU referent cohort. Justification for the development of the Expansion-ICUs in the operating room was multifactorial and included limited number of traditional ICU ventilators (thus necessitating the use of anaesthesia machines as ventilators), the possible need to use a single ventilator for more than one patient (“split-ventilator” strategy), and limited number of critical care staff (physicians and nurses). Staffing these additional beds was accomplished with physicians (attending faculty, residents) from the Departments of Anesthesiology and Surgery, and perioperative nursing staff, including operating room and postoperative care nurses, with respiratory/ventilator support provided by Certified Registered Nurse Anaesthetists (CRNAs) [15, 16]. Staff were redeployed based on request and volunteerism to work in COVID ICU’s with schedules released two weeks at a time [15].

To provide data-based support for the rapid development and deployment of critical care resources during an evolving global pandemic, we performed a retrospective chart review of care characteristics between patients in the standard and non-standard Expansion-ICU setting during the initial phase of COVID-19 crisis at an academic medical center in NYC.

## Methods

### Study design

After determination by the Weill Cornell Medicine IRB that this study was exempt from review (Category 4; Protocol 20–04,021,958), we performed a retrospective observational chart review of COVID-19 patients admitted to an ICU between March 3^rd^ to May 19^th^, 2020. All patients included for analysis were adults (age ≥ 18 years) and had laboratory-confirmed SARS-CoV-2 infection (*i.e.,* a positive polymerase chain reaction assay of nasal and pharyngeal swabs) admitted or transferred to NYP-WCMC for ICU level care. Patients who spent any amount of time in an Expansion-ICU comprised our exposure cohort, and patients who required intubation at any point during hospitalization but who were never triaged to an Expansion-ICU comprised our referent cohort (*i.e*., the traditional ICU patients).

Direct admission to an Expansion-ICU was overseen by a critical care intensivist and determined by overall ICU bed availability and the need to stagger admissions to maintain equitable distribution of workload. A handful of patients were selected to move to the Expansion-ICU to provide capacity in the traditional ICUs. These patients were all mechanically ventilated but did not require dialysis. Triage of patients was performed by a central coordinator, a role that rotated among ICU medical directors. The guidelines for placement were: 1) avoidance of multiple admission to the same unit in rapid succession (within 2–3 h) and 2) avoidance of patients requiring or anticipated to require renal replacement therapy as those nurses were not skilled in these areas and the operating rooms did not have easy access to the necessary facilities (water faucet for hemodialysis or a drain to dispose of dialysis effluent). If their needs were escalating, the patients would also be moved to standard ICUs.

### Data collection

Data collection was performed using automated patient data collection and manual chart review of our electronic medical records (EMRs; EPIC—Epic Systems Corporation, Madison, WI; and AllScripts—Allscripts Healthcare Solutions, Chicago, IL). A priori, we planned to examine the characteristics, hospital course, select laboratory results, ICU treatments and interventions, and COVID-19 specific interventions of patients whose care was provided in the Expansion-ICUs compared to critically ill COVID-19 patients who were never cared for in an Expansion-ICU. Various interventions for COVID-19 were retrospectively reviewed as new data were being published during that time for optimal treatments*.* COVID-specific interventions included use of hydroxychloroquine, remdesivir, tocilizumab, sarilumab, or other immunologics. Laboratory results upon admission to the hospital and maximum and minimum data values throughout the hospitalization were recorded in order to capture disease progression and fluctuations in lab values. All data collected were entered into a REDCap database, which is a secure, browser-based, electronic data capture system used in the design of medical research databases [17].

### Statistical analyses

Descriptive statistics for all variables were calculated for the entire patient population and were compared by ICU type referent vs. Expansion-ICU). Time to intubation, hospital length of stay (LoS; in days), ICU LoS (in days), and management within the ICU were compared within ICU types and between groups of patients discharged from the hospital (either to home or subacute rehab) and patients who died during their hospitalization. Patient age (categorized as: 18–44, 45–64, 65–74, 75 + years) was additionally analysed as it differed across ICU types for each disposition status. Continuous variables were compared using the Wilcoxon rank-sum test, and categorical ones were compared using the Chi-squared test or Fisher’s exact test depending on expected cell frequencies. Results are presented as N (percentage) or median [interquartile range] for nonparametric continuous variables. Results reported for each measure are based on calculations of available (*i.e*., non-missing) data; percentages reported are based denominators of counts of non-missing values for the given category. *P*-values were calculated for each test and were subsequently adjusted for the false discovery rate (q-values) based on the distribution of *p*-values within each table. All tests were two-sided, and significance was evaluated at an alpha level of q ≤ 0.05. Analyses were performed using R version 4.0.1 (R Foundation for Statistical Computing, Vienna, Austria; https://www.R-project.org).

## Results

From March 3 to May 19, 2020, 343 patients were admitted into traditional ICUs and 68 were admitted to an Expansion-ICU. Reason for admission to any ICU at NYP-WCMC was COVID-19-related acute hypoxemic respiratory failure requiring mechanical ventilation. Of the 68 patients admitted to an Expansion-ICU, there was incomplete information on 2 patients, resulting in a final sample size of 66. Of these 66 patients, 60 were also in a standard ICU sometime during their hospital stay. Table 1 displays the characteristics of study cohort, overall and comparing the Expansion-ICU cohort to the Referent cohort. For the Expansion-ICU, there were 45 male (68%) and 21 (32%) female patients; the median age (years; IQR) was 62 (51–70). The majority of patients self-identified as White (32%) or Latino (39%); 7 records (11.0%) did not indicate race or ethnicity. Geographically, patients were primarily from the boroughs of Brooklyn (32%), Manhattan (19%), or Queens (19%). Diagnosed comorbidities prior to admission included 37 with hypertension (56%), 20 with diabetes (30%), and 17 with pulmonary disease (26%). Among Expansion-ICU patients, 33 (50%) were obese and 21 (32%) were overweight. Non-smokers made up the majority of the study group (82%). There were no significant differences in baseline covariates between patients admitted to an Expansion-ICU compared to those never admitted to an Expansion-ICU.

**Table 1:** Patient Characteristics. Table 1 compares the characteristics of patients admitted into the non-traditional ICUs created in postanesthesia care unit (PACU) and operating room (OR) areas (Expansion cohort) to patients that were admitted into the traditional ICUs (referent cohort) the COVID-19 pandemic peak of 2020

**Characteristics**	**Overall** *N* = 409	**Expansion** *N* = 66^a^	**Referent** *N* = 343^a^	*P*-value^*^	**q-value**^b^
**Sex**				0.8	> 0.9
Male	286 (70%)	45 (68%)	241 (70%)		
Female	123 (30%)	21 (32%)	102 (30%)		
**BMI, kg/m**^**2**^** (categorical)**				0.2	0.5
Underweight (< 18.5)	6 (1.5%)	0 (0%)	6 (1.8%)		
Normal Weight (18.5 – 24.9)	104 (25%)	12 (18%)	92 (27%)		
Overweight (25.0 – 29.9)	138 (34%)	21 (32%)	117 (34%)		
Obese (30.0 – 39.9)	160 (39%)	33 (50%)	127 (37%)		
Morbidly Obese (≥ 40.0)	0 (0%)	0 (0%)	0 (0%)		
Missing	1	0	1		
**Age, years (continuous)**	66 (53, 73)	62 (51, 70)	67 (54, 74)	0.045	0.3
**Race**				0.070	0.4
Asian	69 (17%)	5 (7.6%)	64 (19%)		
Black	35 (8.6%)	4 (6.1%)	31 (9.0%)		
Latino	108 (26%)	26 (39%)	82 (24%)		
Not Specified	55 (13%)	7 (11%)	48 (14%)		
Other	18 (4.4%)	3 (4.5%)	15 (4.4%)		
White	124 (30%)	21 (32%)	103 (30%)		
**Comorbidities**^c^				0.9	> 0.9
Diabetes Mellitus	135 (33%)	20 (30%)	115 (34%)	0.7	> 0.9
Hypertension	236 (58%)	37 (56%)	199 (58%)	0.9	> 0.9
CAD	72 (18%)	11 (17%)	61 (18%)	> 0.9	> 0.9
Heart Failure	27 (6.6%)	5 (7.6%)	22 (6.4%)	0.8	> 0.9
CVA	26 (6.4%)	6 (9.1%)	20 (5.8%)	0.4	0.7
COPD	26 (6.4%)	1 (1.5%)	25 (7.3%)	0.10	0.4
Reactive Airway	42 (10%)	9 (14%)	33 (9.6%)	0.4	0.7
Interstitial Lung Disease	3 (0.7%)	1 (1.5%)	2 (0.6%)	0.4	0.7
Other Pulmonary Disease	90 (22%)	17 (26%)	73 (21%)	0.5	0.8
OSA	18 (4.4%)	5 (7.6%)	13 (3.8%)	0.2	0.5
CKD	37 (9.0%)	1 (1.5%)	36 (10%)	0.036	0.3
**Smoking**				0.13	0.4
Current smoker	12 (2.9%)	2 (3.0%)	10 (2.9%)		
Non-smoker	297 (73%)	54 (82%)	243 (71%)		
Past smoker	100 (24%)	10 (15%)	90 (26%)		
**Medications**^d^				> 0.9	> 0.9
ACE inhibitor	119 (29%)	20 (30%)	99 (29%)	> 0.9	> 0.9
Oral steroids	33 (8.1%)	3 (4.5%)	30 (8.7%)	0.4	0.7
Inhaled steroids	26 (6.4%)	5 (7.6%)	21 (6.1%)	0.6	0.8
**Time to Admission to ICU**					
Days	1.6 (0.3, 4.3)	0.3 (0.2, 3.7)	1.9 (0.5, 4.5)	< 0.001	< 0.001
Missing	45	0	45		

^a^ Statistics presented: n (%); median (IQR)

^b^ False discovery rate correction for multiple testing

^c^ *CAD* Coronary Artery Disease, *CVA* Cerebrovascular Accident (*i.e*., stroke), *COPD* Chronic Obstructive Pulmonary Disease, *OSA* Obstructive Sleep Apnoea, *CKD* Chronic Kidney Disease

^d^ Home medications taken prior to hospital admission; ACE inhibitor, angiotensin-converting enzyme inhibitor

^*^ Statistical tests performed: chi-square test of independence; Wilcoxon rank-sum test; Fisher's exact test

Laboratory results were collected when available for patients 24 h upon first admission to standard ICU (for referent cohort) or admission to Expansion-ICU (Table 2). Upon first ICU admission, the Expansion-ICU cohort had significantly lower levels of ferritin (1009 [530, 1464] vs.1446 [87, 2000], q = 0.022), lactate dehydrogenase (LDH) (476 [356, 576] vs 572 [456, 801], q = 0.007), and WBC count (9.1 [6.2, 11.5] vs 11.1 [7.4, 15.1], q = 0.022); in contrast, lymphocyte counts were higher in the Expansion cohort [median (IQR): 10 (5, 15), *n* = 48 vs 7 (4, 12), *n* = 246 in the Referent cohort, *P* = 0.045]. In Expansion ICU patients, the median D-Dimer level was 1,098 (IQR: 631, 2,805; *n* = 24), which was not significantly different (*P* = 0.4) from that observed in the Referent ICU population [median (IQR): 1,749 (641, 4382); *n* = 158].

**Table 2:** Lab Values. Table 2 looks at lab values within 24 h of admittance to a traditional ICU (first admissions only) for the referent cohort and admittance to Expansion ICUs for the Expansion cohort. *p*-values and q-values were generated to examine differences in mean lab values for both cohorts

**Characteristic**		**Referent (*n* = 290)**	**Expansion (*n* = 63)**		*P*-value^*^	**q-value**^a^
	**N**	**Median (IQR)**	**N**	**Median (IQR)**		
**Albumin Level**	266	2.20 (1.90, 2.70)	57	2.40 (1.80, 2.90)	0.8	0.8
**C-Reactive Protein**	153	20 (12, 26)	40	18 (9, 26)	0.2	0.3
**D-Dimer**	158	1,749 (641, 4382)	24	1,098 (631, 2,805)	0.4	0.7
**Ferritin Level**	137	1,446 (807, 2,000)	42	1,009 (530, 1,464)	0.006	0.022
**Haematocrit**	284	36 (31, 40)	53	36 (31, 41)	0.8	0.8
**Haemoglobin**	284	11.95 (10.30, 13.10)	53	12.10 (10.50, 13.40)	0.7	0.8
**LDH**^b^	167	572 (456, 801)	44	476 (356, 567)	< 0.001	0.007
**Lymphocyte**^c^	246	7 (4, 12)	48	10 (5, 15)	0.045	0.12
**Procalcitonin**	155	1 (0, 2)	45	0 (0, 2)	0.085	0.2
**White Blood Cell**	284	11.1 (7.4, 15.1)	53	9.1 (6.2, 11.5)	0.006	0.022
**Interleukin 6**^d^	4	62 (11, 120)	1	7 (7, 7)	0.8	0.8

^a^ False discovery rate correction for multiple testing

^b^ Lactate dehydrogenase

^c^ Automated measurement

^d^ Multi-analyte fluorescent detection (MAFD) method

^*^ Statistical tests performed: Wilcoxon rank-sum test

There were statistically significant differences for time to intubation, hospital LoS, ICU LoS, and time on a ventilator between referent and Expansion cohorts (Table 3). Among discharged patients, the referent cohort had higher median time to intubation (1.88 [0.66, 4.90] vs 1.14 [0.51, 2.80] days, q = 0.039), but lower median time than the Expansion cohort for hospital LoS (40 [22,62] vs 49 [40, 69], q = 0.002), ICU LoS (17 [9,30] vs 32 [25,50] days, q < 0.001), and length of time on a ventilator (17 [10,33] 33 [23,34], q < 0.001). Of note, time to intubation was calculated from admission to the emergency department (ED; or transfer in date) to intubation; however, there were many patients that were intubated before ED admission or transfer in date, giving a negative time to intubation—these patients were not included. Consequently, this variable is limited to patients with a positive time to intubation. For the patients who died, the only significant difference between the groups was that the Expansion cohort had a higher median ICU LoS (25 [16,26] vs. 12 [7,20] days, q = 0.037) and ventilatory length (30 [19,32] vs. 13 [5,20] days, q = 0.015).

**Table 3:** Hospital outcomes

**Characteristic**		**Referent (*n* = 343)** **Median (IQR)**		**Expansion (*n* = 66)** **Median (IQR)**	*P*-value^*^	**q-value**^a^
**Overall – Hospital Outcomes**						
**Time to Intubation (Days)**		2.0 (0.6, 4.9)		1.1 (0.5, 2.7)	0.014	0.014
Missing		93		22		
**Hospital LOS (Days**		27 (15, 50)		45 (30, 65)	< 0.001	< 0.001
**ICU LOS (Days)**		15 (8, 26)		30 (24, 43)	< 0.001	< 0.001
Missing		45		0		
**Vent Length (Days)**		16 (8, 29)		32 (22, 41)	< 0.001	< 0.001
**Discharged – Hospital Outcomes**						
**Time to Intubation (Days)**	1.88 (0.66, 4.90)			1.14 (0.51, 2.80)	0.039	0.039
Missing	65			12		
**Hospital LOS (Days**	40 (22, 62)			49 (40, 69)	0.002	0.002
**ICU LOS (Days)**	17 (9, 30)			32 (25, 50)	< 0.001	< 0.001
Missing	26			0		
**Vent Length (Days)**	17 (10, 33)			33 (23, 43)	< 0.001	< 0.001
**Died – Hospital Outcomes**						
**Time to Intubation (Days)**			2.5 (0.6, 4.7)	0.9 (0.6, 1.2)	0.3	0.3
Missing			28	10		
**Hospital LOS (Days**			16 (8, 25)	23 (16, 27)	0.13	0.2
**ICU LOS (Days)**			12 (7, 20)	25 (16, 26)	0.018	0.037
Missing			19	0		
**Vent Length (Days)**			13 (5, 20)	30 (19, 32)	0.004	0.015

^a^ False discovery rate correction for multiple testing

^*^ Statistical tests performed: Wilcoxon rank-sum test

Management aspects of patients during their ICU stay is in Table 4. The use of vasopressors and prone positioning between the two cohorts was similar regardless of patient disposition. For patients that were discharged, the Expansion-ICU cohort had statistically significantly higher rates of use of propofol (85% vs 67%, q = 0.025), dexmedetomidine (83% vs 42%, q < 0.001), and ketamine (11% vs 2.3%, q = 0.025). For patients who died, there were no statistically significant differences in treatment.

**Table 4:** Medications. Note that vasopressors, hydroxychloroquine, remdesivir, tocilizumab, steroids, and prone positioning were examined at any point during hospitalization. Ketamine, dexmedetomidine, and propofol were examined at any point during traditional ICU stay for the referent cohort and at any point during Expansion ICU stay for the Expansion ICU cohort

**Characteristic**	**Expansion, *N* = 66**^a^	**Referent, *N* = 343**^a^	*P*-value^*^	**q-value**^b^
**Overall—Medications**				
Propofol	57 (86%)	212 (62%)	< 0.001	< 0.001
Dexmedetomidine	50 (76%)	122 (36%)	< 0.001	< 0.001
Ketamine	7 (11%)	10 (2.9%)	0.011	0.032
Vasopressors	66 (100%)	325 (95%)	0.092	0.2
Hydroxychloroquine	1 (92%)	298 (87%)	0.2	0.3
Remdesivir	8 (12%)	45 (13%)	0.8	> 0.9
Tocilizumab	13 (20%)	48 (14%)	0.2	0.3
Steroids (any route)	50 (76%)	227 (66%)	0.2	0.3
**Overall—Prone Positioning**^c^	30 (45%)	161 (47%)	> 0.9	> 0.9
**Discharged—Medications**				
Propofol	46 (85%)	143 (67%)	0.015	0.044
Dexmedetomidine	45 (83%)	90 (42%)	< 0.001	< 0.001
Ketamine	6 (11%)	5 (2.3%)	0.011	0.044
Vasopressors	54 (100%)	201 (94%)	0.13	0.3
Hydroxychloroquine	50 (93%)	188 (88%)	0.4	0.4
Remdesivir	8 (15%)	34 (16%)	0.8	0.8
Tocilizumab	2 (22%)	32 (15%)	0.2	0.3
Steroids (any route)	47 (78%)	144 (68%)	0.15	0.3
**Discharged—Prone Positioning**^c^	23 (43%)	111 (52%)	0.3	0.4
**Died—Medications**				
Propofol	11 (92%)	69 (53%)	0.023	0.2
Dexmedetomidine	5 (42%)	32 (25%)	0.3	0.9
Ketamine	1 (8.3%)	5 (3.8%)	0.4	> 0.9
Vasopressors	12 (100%)	124 (95%)	> 0.9	> 0.9
Hydroxychloroquine	11 (92%)	110 (85%)	> 0.9	> 0.9
Remdesivir	0 (0%)	11 (8.5%)	0.6	> 0.9
Tocilizumab	1 (8.3%)	16 (12%)	> 0.9	> 0.9
Steroids (any route)	8 (67%)	83 (64%)	> 0.9	> 0.9
**Died—Prone Positioning**^c^	7 (58%)	50 (38%)	0.2	0.9

^a^ Statistics presented: n (%)

^b^ False discovery rate correction for multiple testing

^c^At any point during hospitalization

^*^Statistical tests performed: chi-square test of independence; Fisher's exact test

Disposition data was collected for all patients (Table 5). For the Expansion-ICU cohort, 17 (26%) were discharged home, 31 (47%) were discharged to a subacute rehabilitation facility, and 12 (18%) died in the hospital. The two cohorts had similar rates of discharge to home, but the Expansion cohort had higher rates of discharge to a rehabilitation facility with lower rates of death.

**Table 5:** Disposition Status. Table 5 only considers 403 patients (not 409), as 6 patients did not have disposition status identified prior to study censor

Characteristic	Overall *N* = 403	Expansion *N* = 66	Referent *N* = 337	*P*-value	q-value
**Death**	142 (35%)	12 (18%)	130 (39%)	0.002	0.004
**Rehabilitation**	124 (31%)	31 (47%)	93 (28%)	0.002	0.004
**Home**	106 (26%)	17 (26%)	89 (26%)	> 0.9	> 0.9
**Other**	31 (0.08%)	6 (0.9%)	25 (0.07%)	0.6	0.9

## Discussion

### Expansion-ICU management

Our data suggest that it is logistically feasible, and clinically realistic, to rapidly (< 10 days) convert ORs and PACUs into functional ICUs to accommodate significant numbers of critically ill patients. We found no statistically significant differences in patient characteristics (Table 1) or in mortality rates and discharge to home between the Expansion and Referent ICU cohorts (Table 5). There were also no differences in use of vasopressors or prone positioning throughout the entire hospital stay of either cohort for deceased patients; there were, however, notable differences in the use of sedative medications (propofol, ketamine, dexmedetomidine) between the two groups (Table 4), and the implications of this are discussed below.

These results suggest that converting ORs into crisis ICUs allowed for an expansion of ICU capacity under emergency conditions which resulted in non-inferior patient outcomes. Importantly, this was achievable using a minimum of critical care trained personnel. In 2020, global ICU mortality was 35% to 41.6% [18–20]. There was, however, marked differences among countries: from 23.4% in Japan [21] to 57—59.6% in Brazil [22, 23]. Among patient receiving mechanical ventilation (and presumably therefore in an ICU), mortality has shown marked variability: United Arab Emirates—20.2% [24], Netherlands – 38% [25], Italy – 51.7% [26], Germany – 52.8% [27], Russia – 65.4% [28], United Kingdom – 69% [29], Mexico -73.7% [30], and Romania 95% [31]. More locally, ICU mortality in New York City among patients requiring mechanical ventilation has been reported as high as 88.1% [32]. The in-hospital mortality of patients treated in an Expansion-ICU herein is concordant with early reported intra-institutional mortality among patients requiring invasive mechanical ventilation (14.6%) [33], and is markedly less than that reported for other hospitals in the NewYork-Presbyterian system (41%) [34]. This is noteworthy as COVID patients in the NYC area have higher levels of comorbidities, longer intubations, and higher rates of kidney injuries compared to other locations [35].

Recent data indicates that ICU patient load dramatically impacts mortality rates, with lower rates of available ICU beds or increased ICU overflow being associated with increased mortality [36–40]. This may be due to lack of resources and personnel that occurs during times of high clinical burdens, with differing opinions on whether the use of time-dependent changes in clinical practice influenced those outcomes [38, 40]. Taccone et al. also found that the proportion of ICU beds available and the number of newly created ICU beds were each independent risk factors for mortality [40]. In contrast, we found that not only were our mortality rates lower than those of similar hospitals, but our ICU load and expansion ICU areas were not associated with inferior outcomes (Table 5). Of note, however, patients in the Expansion-ICU were more likely to be discharged to a subacute rehabilitation facility than those in the Referent ICU (Table 5); this may reflect the fact that the Expansion-ICU discharged cohort had an ICU LoS that was twice as long as those in the Referent ICU [32 (25, 50) vs 17 (9, 30) days, median (IQR)] and as well as time spent requiring mechanical ventilation [33 (23, 43) vs 17 (10, 33) days, median (IQR)] (Table 3). Prolonged LoS in an ICU is associated with ICU-acquired weakness (“deconditioning”) which can result in profound functional impairment [41], and so the higher incidence of discharge to rehabilitation facilities in the Expansion-ICU population might reasonably be expected. Our results are a clear demonstration that “repurposing” of resources can in fact provide safe and effective care, and support in principle the approach advocated by Diaz et al. for repurposing pediatric ICUs to adult critical care units [42].

We also address the limitations of other studies by providing comparisons of patient characteristics, treatments, and outcomes across different settings. There were a few differences in hospital management for deceased patients between the Referent and Expansion-ICUs (Table 3). The Expansion cohort had a significantly higher ICU LoS and time on ventilator in deceased patients. Those differences could reflect, in part, disease progression and corresponding care-escalation requirements; for example, if a patient required renal replacement therapy (a predictor of disease severity and mortality [33, 34, 43–46]), they would have been transferred to a traditional ICU.

There were some differences in laboratory findings (Table 2). The Expansion-ICU cohort had significantly lower median levels of ferritin, LDH, and white blood cell count (WBC) within the first 24 h of admission to an ICU. In contrast, increased procalcitonin, C-reactive protein, IL-6, ferritin, LDH, and D-dimer levels associated with severe or fatal COVID-19 infections (> 1 μg•mL^−1^) were observed, consistent with prior reports [43, 46–54]. A recent meta-analysis found that compared to patients discharged from the hospital, those that died had higher WBC, ferritin, C-reactive protein, D-dimer, LDH, and IL-6 levels with decreased levels of lymphocytes, hemoglobin, and albumin compared to those that were discharged from the hospital [50]. While the lower levels of ferritin, LDH, and WBC in the Expansion cohort may point to a less severe disease progression, other lab results were similar between cohorts suggesting that the overall severity of illness was comparable.

When considering the co-morbidity of the two groups, the incidence of hypertension, diabetes, or obesity as the most reported comorbidities in both cohorts was comparable to that reported within and outside of the NewYork-Presbyterian system [33, 34, 43–48, 53]. The high rates of cardiovascular disease, pulmonary disease, older age, elevated IL-6 and D-dimer levels that we observed in our cohorts (Tables 1–2) are common features in COVID-19 patients, and are potential predictors of mortality within the wider NYC cohort [34] and elsewhere [44–46, 53, 55]. Previous studies have demonstrated that use of any vasopressors, particularly for extended time periods, was associated with disease severity or mortality [33, 43]. In contrast, however, we found no significant difference in disposition between patients who did and did not receive vasopressors.

Prone positioning has been reported to improve oxygenation in spontaneously breathing non-intubated patients with hypoxemic acute respiratory failure [56] as well as in patients with acute respiratory failure in the setting of COVID-19 respiratory failure [57]. Other studies have also suggested benefits of prone positioning for ARDS and COVID-19, but with its benefits often limited to early use in patients not requiring mechanical ventilation [58–61]. The apparent lack of benefit of proning reported here suggests that its application late in the course of COVID-19 respiratory failure (*i.e*., once invasive mechanical ventilation has been initiated) is not indicated. Whether this is true requires an appropriately designed prospective trial.

There were several differences in hospital management for discharged patients between the Expansion and standard ICU cohorts. While vasopressors and prone positioning remained similar in the discharged group as with the deceased group, the Expansion-ICU cohort had significantly higher rates of use of propofol, dexmedetomidine, and ketamine. Dexmedetomidine administration is associated with improved oxygenation in morbidly obese patients with restrictive lung disease compared to a placebo group [62]. Of direct relevance here, dexmedetomidine, when administered to adult patients with COVID-19 who were admitted to an ICU and required sedation, was associated with a significant increase in the P_a_O_2_/FiO_2_ (PF) ratio 4–12 h following dexmedetomidine administration (PF at baseline; 17 ± 6 vs 21 ± 5 at 6 h, *P* < 0.001) [63]. Critically, dexmedetomidine administration in a different cohort of patients with COVID-19 who required invasive mechanical ventilation had significantly lower 28-day mortality than those who did not receive it (respectively, 27.0% vs 64.5%, relative risk reduction 58.2%, 95% confidence interval 42.4–69.6) [64]. The observed survival benefit in patients who received dexmedetomidine is consistent with our results wherein dexmedetomidine administration was administered more often to patients who were discharged from the hospital as compared to those who died (Table 4). The mechanism(s) through which dexmedetomidine might confer a survival benefit are not known with certainty, but may include: reduced agitation and increased ventilator compliance, enhanced hypoxic pulmonary vasoconstriction (HPVC), and improvement in the ventilation/perfusion ratio ([64] and references therein). Thus, the higher rate of dexmedetomidine administration in the Expansion-ICU cohort may have may have had a beneficial effect on their overall outcomes.

### Limitations

Our findings are associative and will require additional research to define their value in relation to the COVID-19 pandemic. With a much smaller Expansion-ICU cohort compared to the Referent cohort, a larger dataset may have been helpful for detecting small differences in therapeutic interventions between the two groups. As with many retrospective studies, the analyses are only as reliable as the available data, and documentation gaps in the EMRs hampered our ability to perform analyses. Similarly, the study was limited by the fact that some patients were transferred to our care from outside hospitals, and we were unable to obtain full laboratory results and management interventions prior to arrival. Within-hospital transfer between units occurred as well, either due to space limitations or as warranted by disease severity, so this offers an additional confounding variable when analysing patient outcomes between cohorts. Finally, our capacity to rapidly expand services in a large, urban, tertiary care medical center may not be generalizable to smaller hospitals with fewer resources.

## Conclusions

Rapidly expanding ICU capacity for the care of critically ill patients during a surge in need in the midst of a pandemic is feasible. If appropriate resources – staff, material, space – are immediately available, such care can be provided safely and effectively. In the context of a surge in infections and increase in hospitalization and ICU admission rates arising from the SARS-CoV-2 Delta variant, these results provide support for the continued deployment of rapid Expansion-ICUs.

## Data Availability

There are legal and ethical restrictions on data sharing because the Institutional Review Board of Weill Cornell Medicine did not approve public data deposition. The data set used for this study constitutes sensitive patient information extracted from the electronic health record. Accordingly, it is subject to federal legislation that limits our ability to disclose it to the public. A de-identified version of the dataset underlying these findings can be requested upon reasonable request from Dr. Peter Goldstein (pag2014@med.cornell.edu).
